# Virally-initiated pain states: phenotypes, mechanisms, and future directions

**DOI:** 10.3389/fpain.2025.1527106

**Published:** 2025-01-28

**Authors:** Sara A. Dochnal, Steven P. Cohen, Mark R. Hutchinson, Yury I. Miller, Tony L. Yaksh

**Affiliations:** ^1^ Department of Anesthesiology, University of California, San Diego, CA, United States; ^2^ Department of Anesthesiology, Northwestern University Feinberg School of Medicine, Chicago, IL, United States; ^3^ School of Biomedicine, University of Adelaide, Adelaide, SA, Australia; ^4^ Institute for Photonics and Advanced Sensing, University of Adelaide, Adelaide, SA, Australia; ^5^ Davies Livestock Research Centre, University of Adelaide, Adelaide, SA, Australia; ^6^ Department of Medicine, University of California, San Diego, CA, United States

**Keywords:** neuroimmune activation, viral infection, post-herpetic neuralgia, Long COVID, influenza, neurotropic infection and antiviral therapy, microorganism

## Abstract

The recent SARS-CoV-2 pandemic has underscored the significance of viral infections, affecting billions of lives and costing trillions of dollars globally. Even beyond SARS-CoV-2, common infections with viruses like influenza, HIV, and herpesviruses have profound impacts beyond their typical manifestations, often triggering acute and chronic pain syndromes that can be life-altering. These virally induced pain states can arise through direct viral replication within neurons, or indirectly, via immune responses to infection in both the contexts of afferent signaling in the dorsal root ganglion (DRG) or subsequent higher order integration in intracranial systems. Varicella-zoster virus (VZV), influenza virus, and SARS-CoV-2 each provide a unique lens through which to examine the interplay between viral activity and pain. This perspective paper is not meant to be an exhaustive review of virally-induced neuropathic pain states. It seeks to explore curated aspects of the complexities of these pain states, identify research gaps, and suggest solutions using nanoscale molecular understanding and psychoneuroimmunological and biopsychosocial frameworks. Each subheading is accompanied by a list of related issues for study which we think will lead to advances in our understanding of the vexing pain phenotype associated with viral infection.

## 1 Heterogeneity of viral-induced pain states

Virally-associated pain states exhibit a wide variety of presentations across and within viral families, whereby the patient demonstrates varying degrees of allodynia hyperalgesia, paresthesias, and dysesthesias. These pain phenotypes can manifest immediately following infection and persist in the presence of a viral titer, result in changes which persist after a loss of viral titer, or they may present after a pain-free interval. The latter may occur because the virus goes dormant and reactivates after a latent period, or because the initiating event (e.g., central sensitization) takes time to reach the threshold of perception.

i) Influenza virus characteristically produces an acute infection within the respiratory tract. By 2–3 days post-infection, pain presents in the form of sore throat and headache (frontal or retro-orbital), as well as a whole-body pain referred to the musculature (e.g., myalgias) and the joints (arthralgia) (1). Symptoms typically persist until 7–10 days post-infection, corresponding with the clearance of virus. However, Influenza has also been associated with post-viral chronic pain conditions, including post-viral myositis (2, 3), arthralgias (4), and fibromyalgia (5).
ii) SARS-CoV-2 infection initiates an acute phase, wherein the patient may experience myalgias, arthralgias, sore throat, abdominal pain, chest pain, and headache 2–14 days following viral exposure (6, 7). Following initial infection, Long COVID/post-COVID-19 syndrome may develop, a condition defined by the World Health Organization (WHO) as persistent or new symptoms, including joint and chest pain, myalgias, headache, and generalized full body pain which persist for at least 2 months with no other explanation (8, 9).
iii) Alphaherpesvirus varicella zoster virus (VZV) can result in a body-wide blistering skin rash (chickenpox) two weeks following exposure, characterized by intense pruritus over a period of a week (10, 11). The somatic sensation resolves alongside the blistering outbreak, corresponding to the immune clearance of actively replicating virus (12). Reactivation of latent VZV, characteristically during old age and coincident with decline in immunity (13, 14), triggers herpes zoster/shingles, a syndrome which acutely presents as a dermatomal rash in the form of throbbing, stabbing, burning, and pruritus (11, 12). Following the resolution of zoster rashes and viral replication, patients may experience postherpetic neuralgia (PHN), a pain condition lasting more than 3 months past onset. PHN presents in the form of allodynia and hyperalgesia, as well as continuous or intermittent burning, stabbing, shooting, or throbbing referred to the dermatomal distribution of the scarring (10, 12, 15, 16).

The above commentary points to the complexity of the pain phenotypes and profiles that occur with these three representative viral infections. Under the circumstances, it is worthwhile to consider these phenotypes in light of the current thinking regarding the mechanistic classification of pain phenotypes, those that are: nociceptive (e.g., acute, high intensity stimuli and events secondary to tissue injury and inflammation), neuropathic (e.g., secondary to injury of the nerve reflecting axotomy, demyelination, neuronal death), and/or nociplastic (e.g., pain that arises from and is sustained by altered central process leading to activation of nociceptive circuitry despite the absence of evident tissue damage) (17). Given their persistence and behavioral characteristics, the primary mechanistic focus on the sequalae of viral infection is often set on neuropathic pain phenotypes (18). However, many virally-mediated states, such as post-viral chronic pain conditions associated with influenza, as well as Long COVID syndrome, are characterized by long-lasting, non-dermatomally distributed arthralgias and myalgias, suggesting the existence of mechanisms which potentially drive nociplastic actions which are not restricted to generating a neuropathic phenotype. One of the most common virally-mediated pain conditions, herpes zoster or shingles, is a combined phenotype that consists of nociceptive (e.g., blistering lesions on the skin) and neuropathic pain (i.e., DRG and spinal nerve root or cranial nerve involvement).

### 1.1 Topical issues

i) Detailed psychophysical and temporal characterization of human and animal virally-mediated pain states (e.g., dermatomal vs. non dermatomal, joint pain);
ii) Clinical studies on the efficacy of *analgesic* interventions in acute and chronic viral pain states.

## 2 How does viral infection induce changes in cellular function?

The introduction of a virus into an organism has three consequences impacting system function.

i) *Viral particles in the extracellular space or on the cell surface lead to the activation of innate and adaptive host immunity involving populations of natural killer (NK) cells, macrophages, and T and B lymphocytes.* Immune activation leads to the secretion of humoral products like cytokines, chemokines, and antibodies to viral epitopes that impact local cell populations through afferent axons and distal cell populations *via* vascular uptake. From a psychoneuroimmunological perspective, these cytokines—such as TNFα, IL1β, and IL6—play a critical role in modulating pain by directly sensitizing nociceptive neurons and indirectly influencing pain through neuroimmune interactions (19, 20). As the DRG lies outside the blood brain barrier, circulating factors can gain access to the DRG cell systems, from sacral to trigeminal, through the DRG-vascular barrier (21), which can generate ectopic activity and enhanced afferent traffic through the complement of neuronal and nonneuronal cells (22). The concept of neuroimmune crosstalk is central here, as this interaction between immune cells and neurons contributes to the development and persistence of pain, highlighting the importance of immune-driven changes in neuronal excitability in virally-induced pain states.

Influenza infection may alter nociception through both its robust local and systemic inflammatory responses, with increases in circulating lipid mediators and cytokines often referred to as a “cytokine storm” (23, 24). Many immune cells can be infected or activated by Influenza, including mast cells (25), NK cells (26), dendritic cells (27), ILC2, and interstitial macrophages (28) which litter peripheral neuroepithelial junctions and release a variety of cytokines, chemokines, and lipid mediators. Neuroimmune crosstalk, facilitated by these inflammatory signals, plays a crucial role in regulating neuronal responses to infection, but presently there is a lack of information on how this local inflammatory environment directly impacts peripheral terminal neuron depolarization or hyper-excitability in the context of Influenza. Recent evidence has demonstrated that neuroimmune crosstalk does occur through these neuroepithelial junctions during influenza infection and that it regulates neurological behavior. GABA A receptor 1 (GABRA1) in the cell bodies of the glossopharyngeal nerve, which projects to the nasopharynx, senses secreted PGE2 through the PGE2 receptor 3 (EP3) following influenza infection (29). Through the ablation of GABRA1+ neurons or deletion of EP3 receptors in these neurons, symptoms of reduced food and water intake and impaired exercise capacity during early influenza infection can be eliminated, although allodynia or algesia was not measured in this study. This illustrates the importance of neuroimmune signaling in influencing both sensory and non-sensory behaviors during viral infections. Future research should focus on assessing the impact of these immune-neuronal interactions on nociceptive processing and pain perception to better understand their role in virally-induced pain states.

Influenza infection can also lead to a robust immune response in the CNS mediated by proinflammatory cytokines and chemokines and subsequent reactivity in microglia (30–35). The reactive state in microglia induced by these inflammatory mediators plays a key role in shaping the pain response and can lead to long-lasting alterations in neuronal function (36, 37). Studies on the effects of influenza viruses on microglia suggest that infection likely induces a priming state in microglia, which could have profound consequences on the regulatory role that microglia play in local neuronal excitability in the brain and spinal systems (33–35). Primed microglia are more sensitive to milder stimuli, wherein a second stimulus/hit leads to an exacerbated response compared to the first stimulus/hit, akin to temporal summation, a phenomenon observed with central sensitization. Priming results in increased immune reactivity to a secondary insult and makes microglia more resistant to negative/regulatory feedback, leading to impaired synaptic plasticity, and neurodegeneration (36–39). These changes contribute to the persistence and amplification of pain following viral infections. Notably, most of this work focuses on the brain with few references regarding the effect upon spinal or DRG function, though several studies emphasize the potential relevance of these targets. Future research should expand on these areas to provide a more comprehensive understanding of how microglial reactivity contributes to virally-induced pain across different levels of the nervous system.

ii) *Once in the cell, the virus alters the cellular transcriptome to initiate synthesis of proteins necessary for viral replication, which can have ancillary effects on cellular function.* There are increasing examples whereby viruses can lead to persistent changes in cellular signaling, even in reduction or absence of viral titers or where viral advantage is unclear. Viral infection can induce these changes via prominent epigenetic changes in the host cell, a phenomenon commonly observed with the herpesviruses in latently infected cells (40) and Influenza A targets, including immune cells relevant to sensitization like macrophages (41). Innate immune signaling, as occurs through activation of TLR4, can lead to the enlargement of lipid rafts that lead to a persistent aggregation of proinflammatory channels and receptors on the lipid rafts of spinal and DRG glial and afferent soma (22, 42–44). This activation leads to long term changes in cellular excitability, mediated by epigenomic effects. In the case of SARS-CoV-2, viral entry can be mediated by lipid rafts and impaired by depleting membrane cholesterol (45). These data confirm lipid rafts play an important role in viral infection and the ensuing pathophysiology, although their contribution to virally-initiated pain states remains incompletely investigated.

Despite the importance of afferent signaling and the DRG soma, surprisingly few studies have reported on the consequences of their infection with virus e.g., the transcriptome of the DRG neurons, satellite cells, or macrophage/microglia. For example, changes in peripheral neurons in response to influenza infection have gone unreported apart from a single recent study that investigated the impact of neurotropic strain of influenza on vagal sensory neurons at the level of RNA (46). Note that the direct infection of a neuron need not always account for changes in neuronal excitability associated with a pain state. Consider that some strains of influenza are neurotropic, but the capacity of the virus to infect neurons directly does not determine whether a strain causes neurological complications. Both neurotropic and non-neurotropic strains of influenza are associated with pain and neurologic effects, suggesting nociception is mediated independently of a virus' ability to infect a neuron (30, 47, 48). This may occur through immune modulation which then acts to sensitize neurons or through direct infection and reprogramming of non-neuronal cells which may indirectly contribute to sensitization.

iii) *Viral replication initiates trophic responses by the infected cell secondary to the metabolic stress resulting from proliferation of virus, which may lead to damage or cell death.* These responses are relevant in that such impairment in neuronal function is closely linked with the development of a persistent neuropathic pain state. In the scenario of VZV infection, there is substantial evidence that infection causes neuronal damage within the DRG of the PNS and in the CNS. Skin biopsies (49–51) and post-mortem analyses (52) in PHN patients demonstrate a loss of cutaneous innervation and damage to the dorsal horn. In rodent models, infection has been reported to lead to structural changes and mild apoptosis in neurons, as well as increases in neuropathic damage markers including Nav1.3, neuropeptide Y (NPY), and galanin (53, 54). Collectively, these data support a state of neuronal cell damage or death within the ganglion linked to pain states during PHN. Similarly, SARS-CoV-2 infections results in neuronal degeneration in patients (55, 56), with infection being shown to induce neuropathic transcriptomic changes in DRG neurons following acute and chronic infection in a hamster model (57). Influenza infection has been shown to cause a loss of dopaminergic neurons (31, 58) and *in vitro* infection within rodent cortical neurons has resulted in reduced neuronal cell viability (59) although effects of Influenza on DRG neurons remain ominous.

### 2.1 Topical issues

i. Investigation of if and how specific inflammatory factors, and the cells from which they originate, contribute to nociceptive readouts;
ii. Systematic assessment of changes in glial and neuronal function after acute and persistent exposure of cell systems to virus;
iii. Assessment of resulting host transcriptome and phosphoproteome, and neuronal damage and death, following viral infection in neuronal and non-neuronal cell types, even with non-neurotropic viruses that have been found to drive pain states;
iv. Assessment of the effects of viruses in producing long-term changes in pain processing;
v. Assessment of effects of epigenetic and genome modification that promote or prevent a long-term viral effect upon neuraxial cellular processing.

## 3 Access and entry: what factors govern the viral targeting of cell types

Consequences for pain differ based on a virus' ability to gain access and entry into cells that modulate pain phenotypes, such as neurons and companion immune cells within the PNS and CNS.

The cellular tropism of the virus at one level depends upon the nature of the mechanisms it employs to gain entry to the cell, such as binding to specific cell surface receptors and/or fusion to epitopes often dependent upon a fusion protein embedded in the protein capsid coating. Binding at specific membrane sites leads to viral particle internalization though the formation of pores and engulfment by endosomes or, on other occasions, direct injections of genetic material. For example, hemagglutinin molecules coating the envelope of Influenza viruses bind to receptors containing sialic acid on the cell surface, resulting in the endocytosis and endosomal entry of viral particles (60). VZV uses a combination of envelope glycoproteins (glycoprotein B, H, and L, referred to as gB, gH, and gL) to facilitate virion binding to several host cell receptors (myelin associated glycoprotein/Siglec4, Mannose-6-phosphate/MPR, insulin degrading enzyme/IDE) and facilitate fusion of the host membrane and virion envelope (61). The spike protein of SARS-CoV-2 binds to the angiotensin-converting enzyme 2 (ACE2) receptor expressed on host cells, and this event is followed by the cleavage of the spike protein by the host protease and viral fusion to enter the host cell (62). These viral proteins and processes enhance the ability of viruses to directly infect neurons, leading to damage or alteration of signaling pathways which alter excitability, as well as amplifying immune responses that also contribute to changes in neuronal excitability. Many of these receptors localize to lipid rafts in the host plasma membrane, and virus entry often depends on the size and integrity of lipid rafts (63, 64).

The ability of a virus to infect certain cell types also depends on the viral capability to traffic to the appropriate location to contact the target cell. VZV directly infects neurons and likely accesses the innervating peripheral nervous system including the DRG through retrograde transport (65), and possibly through a hematogenous route based on data employing a similar virus, the Seneca Valley Virus (SVV) (66). In addition to its effect on peripheral terminals, there is evidence that Influenza viruses can be trafficked by retrograde transport in the afferent component of cranial and somatic nerves (67, 68). This suggests that the virus has access to the DRG neuronal soma and is trafficked to second order synapses in the dorsal horn. Viruses infecting neurons may migrate along the axon to the soma and often beyond to the first order synapse. Influenza may access the CNS through direct infection via the olfactory bulb (69) or by hematogenous spread due to enhanced permeability of brain microvascular endothelial cells following infection (34, 70). How SARS-CoV-2 infiltrates the CNS is not completely clear, but it has been postulated that it travels through the bloodstream, penetrating the brain through the vascular wall and transcytosing through the infected vascular endothelial cells (71) or potentially through infection of the olfactory bulb (72).Therefore, there are several routes by which a virus can access the PNS and CNS, including retrograde transport following direct peripheral terminal access, hematogenous routes, or direct infection into the CNS via the olfactory bulb.

### 3.1 Topical issues

i) Elucidating the mechanisms involved in and manifestations of full viral tropism in neuronal and non-neuronal cells involved in generating and maintaining pain states;
ii) Systematic assessment of viral uptake into DRG neurons, macrophages, and satellite cells after systemic and intrathecal delivery;
iii) Examination of the role of lipid rafts in viral entry into DRG neurons;
iv) Assessment of uptake in the peripheral afferent terminal and virion transport to the DRG and higher order neurons *in vivo* and in culture;
v) Characterization of the transcriptome of the DRG and CNS cellular components after local and systemic delivery;
vi) *In vitro* studies in neuronal and non-neuronal cell types and subpopulations of cell type (e.g., peptidergic vs. non peptidergic afferent soma) in combined cultures on the uptake of virus into neurons and the support cells (e.g., glia, fibroblasts).

## 4 Models for assessing effects of viruses relevant to nociception

Whereas many animal models of pain exist, there are few model systems created to specifically address pain induced by viral infections. A model of influenza-initiated pain was developed in which rodents acquire a similar pain profile in terms of presentation and duration (73). However, this model has not been employed since its introduction and pain analysis is limited to mechanical withdrawal thresholds measured after stimulation using Von Frey filaments (73). The pain phenotype of VZV has been more extensively studied despite major limitations in modeling herpetic pain due to the human-specificity of the virus (74). Although rodent models of VZV-induced acute and persistent pain exist, there is still no model that simulates pain during VZV reactivation from latency (12). Moreover, many VZV models do not accurately replicate the robust inflammatory response observed in humans after infection (12), making this component's contribution to pain difficult to quantify. There is a hamster model of pain following SARS-CoV-2 infection (57), but studies are rare due to the robust safety measures infection with SARS-CoV-2 necessitates. Among the models of virally associated pain that do exist, few if any account for important epidemiological factors that play a role in pain such as sex or age or consider how antivirals or general analgesics may impact pain sensation for a given viral infection.

### 4.1 Topical issues

i) Novel *in vivo* models of infection with emphasis on the temporal expression patterns of the pain phenotype;
ii) Demonstration of the aversive nature of the viral infection on non-evoked pain behavior (e.g CP, activity cycles, running wheels, marble burying);
iii) Assessment of the analgesic pharmacology in models of virus-induced pain states.

## 5 Conclusions

As exemplified through SARS-CoV2, VZV, and influenza, the study of how a virus causes pain is complicated by the heterogeneity of viral pain states and presentations, and has begun dissection through the navigation of the (in)direct effects on (neuronal) cell function, and the mechanisms of entry into the DRG or intracranial systems. This area requires more detailed characterizations of animal models to be used in complement with *in vitro* studies to dissect the molecular, cellular, and neuroimmune-mediated mechanisms of pain initiation and chronification. The viral pain niche is a complicated, emerging field (75, 76) that calls for the interdisciplinary efforts of virologists, neuroscientists, immunologists, and pain researchers, which we hope to attract and inspire with this special topic.

## Data Availability

The original contributions presented in the study are included in the article/Supplementary Material, further inquiries can be directed to the corresponding author.
